# First-Line Treatment of Older Patients with CLL: A New Approach in the Chemo-Free Era

**DOI:** 10.3390/cancers15153859

**Published:** 2023-07-29

**Authors:** Antonio Urso, Francesco Cavazzini, Maria Pia Ballardini, Silvia Gambara, Sara Consolo, Gian Matteo Rigolin, Antonio Cuneo

**Affiliations:** Hematology Unit, University of Ferrara, 44121 Ferrara, Italycvzfnc@unife.it (F.C.); mariapia.ballardini@edu.unife.it (M.P.B.); sara.consolo@edu.unife.it (S.C.);

**Keywords:** chronic lymphocytic leukemia, older patient, Bruton tyrosine kinase, BCL2, cost-effectiveness

## Abstract

**Simple Summary:**

The modern treatment of chronic lymphocytic leukemia (CLL) has dramatically changed thanks to the development of effective mechanism-based drugs, which have proven to be superior to chemoimmunotherapy in all age groups. Because the choice of treatment for older patients largely depends on fitness status rather than chronological age, we aimed to discuss and put into perspective (i) the definition of an older patient, (ii) the efficacy of targeted agents in this patient population, and (iii) the cost-effectiveness of targeted therapy in high-income countries.

**Abstract:**

Bruton tyrosine kinase inhibitors (BTKi) and the BCL2 inhibitor venetoclax, with or without the anti-CD20 monoclonal antibody Obinutuzumab, represent the preferred options for the first-line therapy of CLL because they are more effective and may improve quality of life. However, patient inclusion criteria are heterogeneous across trials designed for older patients, and the identification of CLL-specific parameters identifying unfit patients at risk of developing drug-specific adverse events is required to guide treatment choice. Due to inclusion/exclusion criteria in trials, higher discontinuation rates with BTKi were reported in real-world studies, and registry analyses provided useful information on factors predicting earlier discontinuation in a real-world setting. Though targeted agents were shown to be cost-effective treatments in high-income countries, the out-of-pocket expenses may limit accessibility to these drugs, and the overall expenditure for new drugs in CLL is projected to increase substantially, posing an issue for sustainability. This being said, the choice of a finite-duration treatment based on venetoclax-containing regimens or treatment until progression with BTKi is today possible in high-income countries, and the therapy choice drivers are represented by coexisting medical conditions rather than age, patient expectations, logistics, and sustainability.

## 1. Introduction

Chronic lymphocytic leukemia (CLL) is one of the most frequent types of leukemia, representing 1% of all cancer cases [1]. The reported incidence is 4.7 cases per 100,000, and the probability of developing CLL during a lifetime is 0.6%, with an estimated 207,463 CLL patients living in the United States in 2020 [1]. In 2023, 18,740 new CLL patients were diagnosed in the U.S., and there were 4490 deaths [2]. The median age at diagnosis is around 70 years, with the highest percentage of new cases among people aged 65–74 [1], and an estimated 10-year prevalence of 49.8 people per 100,000 inhabitants was reported in the U.K. [3]. Five-year relative survival has continuously increased during the past decades [4], with a 5-year relative survival of 88% [1]. Considering the increasing life expectancy of the general population in many countries and the advances in treatment, it is reasonable to predict that the incidence and prevalence of CLL will increase [5].

While for decades the mainstay of CLL treatment was chemotherapy [6], in 2010, the chemoimmunotherapy (CIT) regimen of fludarabine, cyclophosphamide, and the anti-CD20 monoclonal antibody (MoAb) rituximab (FCR) was shown to improve overall survival (OS) and progression-free survival (PFS) in fit patients as compared with chemotherapy [7]. Furthermore, durable responses were observed with FCR in up to 2/3 cases with favorable genetic profiles, i.e., mutated configuration of the immunoglobulin heavy chain gene (IGHV) and absence of 11q- and TP53 aberrations [8,9]. While FCR became standard of care in young patients, older patients, who are frequently affected by coexistent medical conditions, were shown to benefit from CIT regimens combining an anti-CD20 MoAb with chlorambucil or bendamustine [10,11,12].

More recently, the treatment of CLL dramatically changed following the demonstration of a significant PFS advantage with Bruton tyrosine kinase inhibitors (BTKi) or with the BCL-2 inhibitor venetoclax in combination with anti-CD20 MoAb as compared with CIT [13,14,15,16]. However, while the BTK inhibitor ibrutinib was shown to have an OS advantage over FCR in young patients [13], there is no documented OS advantage with these new agents as compared with CIT using anti-CD20 MoAb with bendamustine or chlorambucil in elderly patients. 

In this review, we summarized and discussed existing evidence on the usage of targeted agents in older patients requiring first-line treatment with particular reference to (i) the definition of an older patient, (ii) the efficacy of targeted agents in this patient population, and (iii) the cost-effectiveness of targeted therapy.

## 2. Literature Search

A literature search was performed to identify studies evaluating the role of targeted agents in the upfront treatment of CLL, covering three areas: clinical trials, real-world data, and cost-effectiveness.

We used PubMed as the search engine, using MeSH-controlled vocabulary as follows: ((“Leukemia, Lymphocytic, Chronic, B-Cell” [Mesh]) AND (“ibrutinib” [Supplementary Concept] OR “acalabrutinib” [Supplementary Concept] OR “zanubrutinib” [Supplementary Concept] OR “venetoclax” [Supplementary Concept])). Citations were restricted using the PubMed age filter (65+ years), and 458 citations were retrieved, spanning a period from Oct 2012 to March 2023. The manuscripts included in our analysis fulfilled the following inclusion criteria: (i) English language; (ii) full-text paper available; (iiia) phase 3 clinical trial, (iiib) real-world study, or (iiic) cost-effectiveness studies; (iv) first-line therapy of CLL; (v) elderly or unfit patient population. Papers reporting efficacy data on CIT were excluded. The Preferred Reporting Items for Systematic Reviews and Meta-Analyses (PRISMA) guidelines [17] were used to report the manuscript selection process (File S1; Supplementary Material).

## 3. Older Patients

Physiological age is determined not only by chronological age, but also by the functional status of each patient, which may take into account the fitness status as assessed by the presence and severity of comorbidities and organ function and by a comprehensive geriatric assessment (CGA) [18]. Therefore, age is not “per se” a criterion for the choice of the intensity of a given treatment in CLL. Indeed, a cumulative illness rating scale (CIRS) score of ≥6 [19] and/or a creatinine clearance (CrCl) of ≤70 mL/min were adopted to identify unfit patients to be enrolled in clinical trials of the German Cooperative Study Group (GCLLSG) [20], whereas in other trials, an age cut-off of ≥65 years or the presence of existing medical conditions were adopted as the main inclusion criteria [15,21,22,23,24]. Thus, the definition of an older patient ineligible for a fludarabine-based regimen in CLL was heterogeneous across published clinical trials, and the impact of coexisting medical conditions or CGA may represent more appropriate tools than age to identify patients eligible for intensive treatment.

### 3.1. Impact of Comorbidities

The number of coexisting medical conditions in patients with cancer increases with age, and at CLL diagnosis, up to 93% of patients have at least one comorbidity [25]. Patients in the 65–74 age range carry a median of 3.6 comorbidities [26]. Comorbidities can be quantified with various scores, such as the cumulative illness rating scale (CIRS) [19], the Carlson comorbidity index (CCI) [27], and the National Cancer Institute (NCI) comorbidity index [28]. 

A CIRS score of ≥6 at diagnosis was found to be associated with a shorter OS independent of CLL-IPI in a cohort of 335 untreated CLL patients treated at a single institution [29]. Interestingly, a retrospective analysis of the Danish CLL register demonstrated that comorbidities at diagnosis were associated with CLL-related mortality at multivariable analysis, with 38% of comorbid patients having died from CLL-related causes at a median follow-up of 3.3 years [30]. In a prospective U.S. cohort study [25], the impact of comorbidities on mortality was assessed in 1143 patients with newly diagnosed CLL. After a median follow-up of 6 years, 225 patients (20%) died, and the causes of death were as follows: CLL progression in 46% of the cases; comorbid health conditions in 27% of cases; infection in 8% of cases; and other cancers in 19% of cases. At multivariate analysis, however, the CCI score was significantly associated with non-CLL-specific mortality but not with CLL-related mortality. The younger age of the U.S. cohort vs. the Danish cohort (63 vs. 71 years) may account for this observation. Because CLL or its complications are the leading causes of mortality, regardless of CCI score or the number of comorbidities, CLL-directed therapies that can be used on elderly and comorbid patients are needed [25,30].

The role of comorbidities as a prognostic factor was evaluated not only in newly diagnosed patients but also at the time of treatment. At the time of progression requiring treatment with CIT, a simplified, CLL-specific comorbidity index was shown to correlate with survival in CLL [31]. For patients treated with ibrutinib, the CIRS score appeared to have a negative impact on OS in 145 patients (80% relapsed/refractory) evaluated retrospectively by Gordon et al. [32], whereas in a similar series including 712 patients (75% relapsed/refractory), the CIRS score was predictive of shorter EFS and PFS but not of OS [33].

Applying a machine-learning algorithm to a CLL patient cohort, the most important comorbidities were identified in order to generate the CLL comorbidity index (CLL-CI), which represents a simplified and more specific comorbidity score [34]. The CLL-CI stratified the patients into three risk groups based on vascular, endocrine, and gastrointestinal comorbidities at the time of treatment initiation. The favorable, intermediate, and high-risk groups were associated with statistically significant differences in terms of EFS and OS. The CCI-CI was applied at the time of diagnosis and at the time of first-line treatment in a large cohort of CLL patients in the Danish CLL register. In this analysis, the authors were able to demonstrate that CLL-CI was independently predictive of TTFT, EFS, and OS from diagnosis and was associated with shorter EFS and OS from the time of first therapy [35].

### 3.2. Impact of CGA

CGA represents an accurate evaluation of physiological age [18]. CGA explores multiple domains, including functional status, physical health, social and environmental issues, and psychological health [36]. Although there is an association between PS and CGA, the latter appears to be an independent factor capable of adding information on the functional status of elderly patients with cancer, including patients with a good PS [37].

Applying the CGA to 75 older patients enrolled in the CLL9 trial, 19%, 63%, 49%, and 36% of the patients showed an impairment of instrumental activities of daily living (IADLs), physical performance, cognitive ability, or a high burden of comorbidity, respectively [38]. Interestingly, decreased physical and cognitive capacity were predictive of decreased survival.

Because CGA requires a multidisciplinary team and specific assessment tools, it is a time-consuming procedure rarely used in clinical practice or in CLL clinical trials. That said, it is worth noting that CGA may be useful to predict treatment tolerance, OS, and health care utilization, such as hospitalization and emergency room visits [36]. Moreover, CGA can be assessed not only before treatment, but also multiple times in a longitudinal way, thus enabling the treating physician to understand how the patient is tolerating treatment and which domains are most affected by therapy. Thus, CGA may guide supportive interventions, such as those by the physical therapist, dietician, and psychologist [36]. 

Bonanad et al. developed the Geriatric Assessment in Hematology (GAH), which represents a simplified assessment of CGA [39]. The GHA scale was specifically designed for the evaluation of older subjects with different hematological malignancies, 33% of whom were affected by CLL. Interestingly, the mean time to complete the scale was only 12 min [39], and the GAH scale was validated by additional studies [40]. 

Interestingly, a planned analysis of the Alliance trial [21] evaluated the significance of CGA in a population of CLL patients treated upfront with CIT or ibrutinib+/− rituximab, showing that the domains of social activity and nutritional status were significantly associated with PFS and/or OS, regardless of the treatment received. However, no domain was associated with the probability of developing high-grade toxicity or treatment discontinuation among those enrolled in this trial. Moreover, the assessment of some domains was underrepresented (e.g., cognitive impairment) [41].

These data show that, in general, the assessment of comorbidities and CGA may assist the clinician to tailor the intensity of treatment to the needs of the older patient, and that refinement of the tools that assist the clinician in determining the fitness status and tolerability of novel agents represents an area of important investigation [41].

## 4. Data from Trials

Despite CLL being a disease of the elderly with a median age at onset of around 70 years, the majority of published clinical trials until 2010 enrolled patients with a median age between 58 and 64 years [42]. The need for clinical trials specifically designed for older patients was recognized, and the CLL5 trial showed that the purine analog fludarabine was not superior to chlorambucil in older patients [43]. While bendamustine with the anti-CD20 monoclonal antibody rituximab (BR) was shown to be an effective treatment in fit patients > 65 years [11] and guidelines for the usage of bendamustine in CLL were published [12], the CLL 11 trial was specifically designed for elderly and unfit patients. In this randomized phase 3 study, single-agent chlorambucil was compared with chlorambucil associated with the anti-CD20 monoclonal antibody rituximab or the second-generation anti-CD20 obinutuzumab [10]. Chlorambucil plus obitutuzumab (Chlor + O) produced a significant PFS advantage as compared with chlorambucil and rituximab. Furthermore, 37.7% of the patients who received chlorambucil and obinutuzumab attained an undetectable minimal residual disease in the peripheral blood and experienced prolonged PFS, especially in those cases with a favorable genetic profile, i.e., with a mutated configuration of the immunoglobulin gene [10]. Thus, the combination of Chlor + O or the BR regimen became standard treatment regimens for older patients [44].

Following the demonstration of excellent activity in relapsed/refractory CLL [45,46], targeted agents revolutionized the treatment of CLL, including patients with genetically defined high-risk disease [47,48]. Therefore, several randomized phase 3 trials were designed to test the efficacy of targeted agents as compared with standard CIT in treatment-naïve older patients. A summary of salient data for these trials is presented and discussed here.

### 4.1. Bruton Tyrosine Kinase Inhibitors

Five randomized trials showed the superiority of the BTKi ibrutinib, acalabrutinib, and zanubrutinib as compared with chemo(immuno)therapy in previously untreated older patients. The salient efficacy data at the time of the primary pre-planned analyses in these trials are summarized in Table 1, and the incidence of adverse events of clinical interest is reported in Table 2. 

Updated results with longer follow-up were published for these studies. In the RESONATE-2 trial, 269 patients 65 years of age or older were randomized to receive ibrutinib or the chemotherapy agent chlorambucil. Patients between the ages of 65 and 70 years of age had one or more comorbidities that precluded the use of frontline chemoimmunotherapy with FCR. Patients with del17p were excluded. At an extended median follow-up of 7.4 years [51], the experimental arm showed an increased 7-year PFS of 59% vs. 9% [HR 0.154; 95% CI (0.108–0.220)] and an OS benefit despite crossover to ibrutinib at progression in the chlorambucil arm, with a median OS not reached vs. 89 months [HR 0.453, 95% CI (0.276–0.743)]. The benefit of ibrutinib was consistent across all subgroups, and there was no significant difference in PFS in the ibrutinib arm in patients <70 or ≥70 years of age. Ibrutinib was well tolerated, with a median duration of treatment of 74 months and 42% of patients on ibrutinib having up to 8 years of follow-up. The most frequent all-grade adverse events (AEs) with ibrutinib were diarrhea (50%), cough (37%), and fatigue (37%). Most of the ibrutinib-associated AEs decreased over time, with the exception of hypertension, which showed prevalence rates of 25%, 23%, and 25% of patients in years 5–6, 6–7, and 7–8, respectively [51]. Grade ≥ 3 atrial fibrillation (AFib), grade 3 major hemorrhage, and cardiac fatal events occurred, respectively, in 6%, 7%, and 3% of patients in the experimental arm [51]. Grade ≥ 3 infections with ibrutinib occurred in 23% of patients at a median follow-up of 29 months [52]. Ibrutinib was discontinued because of AEs in 24% of patients, and 23% required a dose reduction because of AEs. Noteworthy, greater quality of life (QOL) improvements were recorded with ibrutinib as compared with chlorambucil in the Functional Assessment of Chronic Illness Therapy-Fatigue. However, clinically meaningful improvements, though occurring more frequently with ibrutinib than chlorambucil, did not reach statistical significance [52].

The Alliance trial (A041202) randomized 547 untreated CLL patients 65 years of age or older to receive ibrutinib (I), ibrutinib with rituximab (I + R), or BR [21]. Patients with del(17p) were included. With a median follow-up of 55 months [53], the estimated 48-month PFS was 76% in both I-containing arms as compared with 47% in the BR arm, and 48-month OS estimates were 85% in the I arm, 86% in the I + R arm, and 84% in the BR arm. Adverse events of clinical interest with ibrutinib included all-grade AFib in 19% of the patients, as compared with 6% in the BR arm. All-grade hypertension was recorded in 73% of the patients on ibrutinib and in 54% of the patients on BR. Interestingly, the AE score was higher in the CIT arm for the first six cycles than in the ibrutinib-containing arms, whereas it was lower with BR when comparing the entire duration of assessment. This observation should be interpreted with caution because only unsolicited, treatment-related grade 1–2 and all-cause grade 3–4 AEs were captured for patients in observation after BR [54].

The iLLUMINATE trial compared ibrutinib plus obinutuzumab (I + O) to Chlor + O in 229 patients unsuitable for fludarabine based chemoimmunotherapy because they were older than 65 years or younger with comorbidities, as assessed by a CIRS score ≥ 6 [22]. A clear PFS advantage was documented in the I + O arm as compared with the Chlor + O arm, with an estimated 42-month PFS of 74% vs. 33% and a 75% reduction in the risk of disease progression or death (HR 0.25; 95% CI: 0.16–0.39; *p* < 0.0001). Interestingly, a significant PFS advantage in the I + O arm was also noted among patients with a favorable immunogenetic profile, i.e., with a mutated status of the immunoglobulin heavy chain gene (M-IGHV) (HR: 0.20; 95% CI: 0.07–0.59). Moreover, patients with or without TP53 aberration (del17p or TP53 mutation) had a similar PFS (HR 0.9) in the experimental arm [22]. 

Acalabrutinib is a second-generation BTKi characterized by greater specificity for BTK and fewer off-target effects [55]. 

ELEVATE-TN is a phase III randomized trial that enrolled 535 untreated patients ≥ 65 years of age or younger with a creatinine clearance of 30–69 mL/min or CIRS > 6 [24]. The experimental arms were acalabrutinib with obinutuzumab (A + O) or without (A), and the control arm was Chlor + O. At a median follow-up of 46.9 months [14], a PFS of 87%, 78%, and 25% was reported in the A + O, A, and Chlor + O arms, respectively. The addition of obinutuzumab to acalabrutinib was associated with a significant prolongation of PFS as compared with A alone (*p* = 0.0296). In the subgroup of patients with TP53 aberration (del17p and/or TP53 mutation), the estimated 4-year PFS was 75% in both acalabrutinib-containing arms. IGHV mutational status was not predictive of an inferior PFS in patients treated with A and A + O. Median OS was not reached in all treatment arms, and no survival advantage was observed in the experimental arms as compared with the CIT arm. Acalabrutinib-containing arms were associated with a higher incidence of all-grade headache, diarrhea, fatigue, arthralgia, cough, and upper respiratory tract infection. At a 4-year follow-up, grade ≥ 3 infections occurred in 23.6% of patients in the A + O arm, in 16.2% of patients in the A arm, and in 8.3% of patients in the Chlor + O arm. The incidence of any-grade AFib and hypertension was 3.9%/7.9% and 6.1%/7.3% in patients exposed to A + O and A, respectively, as compared with 0.6%/4.1% in the Chol + O arm. Although cross-trial comparisons should be interpreted with caution, it is worth noting that these data on the incidence of cardiovascular events with acalabrutinib compare favorably with those reported in ibrutinib trials, and that a head-to-head comparison of acalabrutinib and ibrutinib in the relapsed/refractory setting showed a better tolerability profile in the acalabrutinib arm [56]. At a 4-year follow-up, second primary malignancy (SPM), including non-melanoma skin cancer, was reported in 15.7%, 13%, and 4.1% of patients, respectively, treated with A + O, A, and Chlor + O [14]. 

Zanubrutinib is a second-generation BTKi, that was tested in treatment-naïve CLL in the SEQUOIA trial [15]. Patients enrolled were older (>65 years) or younger with comorbidities (CIRS > 6), creatinine clearance < 70 mL/min, a history of severe or frequent infections, which rendered them unsuitable for FCR [15]. Patients without del17p were randomized to receive zanubrutinib (group A) or BR (group B), while patients with del17p were enrolled in the non-randomized group C.

In 479 patients randomized to zanubrutinib or BR, the overall response rate (ORR) was 95% vs. 85%, respectively. The experimental arm showed a significant prolongation of the 24-month PFS (85.5% vs. 69.5%), and a PFS advantage in the subgroup of M-IGHV became apparent in a recent updated report with a median follow-up of 43.7 months [57]. The median OS was not reached in both groups. Grade ≥ 3 AE and discontinuations due to AE were reported more frequently in the BR arm than in the zanubrutinib arm (79.7%/13.7% vs. 52.5%/8.3%). A 5% and 14.2 incidence of major bleeding and hypertension were reported in the zanubrutinib arm at a median follow-up of 26.2 months, and, interestingly, any grade AFib was reported in 3% of the cases in the zanubrutinib arm and in the BR arm [15].

In group C, 109 patients with del17p and a median age of 70 (range 66–74) were treated with zanubrutinib as a single agent. The ORR was 94.5%. The estimated PFS at 18 months was 88.6%, with an OS of 95.1%. Safety data were consistent with those reported in previous studies of zanubrutinib. Clinically relevant AEs were AFib in 2.8% of patients, major bleeding in 5.6% of patients, and no central nervous system events [58]. Other cancers were reported in 13%, 9%, and 22% of patients in groups A, B, and C; it is worth noting that in group C, 10.8% of cancers were basal cell carcinoma of the skin [15].

### 4.2. The BCL-2 Inhibitor Venetoclax

BCL-2, a negative regulator of the mitochondrial pathway of apoptosis, was found to be upregulated in CLL as a consequence of chromosome 13q deletion, causing loss of the negative regulatory miRNA-15a/16-1 [59]. Venetoclax is the first BCL2 inhibitor and was approved by the FDA and EMA for the treatment of CLL following the publication of studies that showed its efficacy in CLL with 17p [60], in relapsed/refractory CLL [61], and in treatment-naïve CLL [20].

The CLL 14 study is a phase III trial that enrolled 432 previously untreated patients with a median age of 72 years. The patients had coexisting comorbidities as defined by CIRS > 6 and/or CrCl < 70 mL/min [20]. The patients were randomized to receive venetoclax and obinutuzumab (V + O) or Chlor + O. In both arms, obinutuzumab was given for six cycles. A higher ORR was obtained with V + O (84.7%, including 49.5% CR) as compared with Chlor + O (71.2%, including 48.1% CR) [16]. At a median follow-up of 65.4 months, PFS was longer in the V + O arm than in the Chlor + O arm ([HR] 0.35 [95% CI 0.26–0.46]), and the estimated PFS rate at 5 years after randomization was 62.6% after V + O and 27.0% after Chlor + O [62]. The PFS benefit provided by venetoclax was independent of IGHV mutational status and TP53 disruption. Interestingly, a longer PFS was observed with V + O as compared with Chlor + O, both in the IGHV unmutated subset (HR 0.25; 95% CI, 0.17 to 0.37; *p* < 0.0001) and in the IGHV mutated subset (HR 0.36; 95% CI, 0.19 to 0.68; *p* = 0.002) [16]. However, it is worth noting that PFS in both arms was shorter in high-risk subsets, as defined by TP53 disruption and unmutated IGHV. No significant difference in OS was detected at the last follow-up [62]. 

Fixed-duration therapy with V + O and Chlor + O produced deep responses with undetectable minimal residual disease (uMRD), which represents a prognostic factor predictive of a longer PFS [10,63].

In CLL 14 MRD was measured in peripheral blood (PB) and bone marrow (BM) by an allele-specific oligonucleotide polymerase chain reaction (ASO-PCR), with a cutoff for uMRD at 10^−4^ [16]. A higher percentage of patients attained an uMRD in PB at the end of treatment (EoT) in the V + O arm (75%) than in the Chlor + O arm (35.2%, *p* < 0.001). Likewise, a higher fraction of patients was shown to attain uMRD in the BM in the V + O arm (56.9%) as compared with the Chlo + O arm (17.1%) (*p* < 0.001). The duration of uMRD status was longer in the V + O arm than in the Chlor + O arm, with a median time to reach a detectable MRD at 10^−2^ of 1259 days vs. 233 days (*p* < 0.0001). Interestingly, univariate and multivariate analysis for MRD conversion by NGS from <10^−4^ at the end of treatment to ≥10^−4^ in the whole cohort showed that age ≥ 75 years had no impact on the duration of uMRD [16].

Treatment was discontinued due to AEs occurring in 16.0% and 15.4% of patients in the V + O arm and the Chlor + O arm, respectively [16]. The most common grade ≥ 3 AE was neutropenia, 52.8% in the former arm vs. 48.1 in the latter arm. With a grade ≥ 3 infection rate of 17.5% with the V + O arm and a 15.0% rate with the Chlor + O arm, the treatment proved to be well tolerated in this elderly patient population, which showed a non-significant increase in the incidence of SPM in the V + O arm (12.7%) as compared with the Chlor + O arm (7.5%) (*p* = 0.074) [62]. A summary of AEs of clinical interest with venetoclax-containing regimens is shown in Table 3.

A pre-specified secondary endpoint of CLL14 was the evaluation of health-related QOL and the burden of CLL-specific symptom severity, based on the European Organization for Research and Treatment of Cancer Quality of Life Questionnaire Core 30 (EORTC QLQ-C30) and the MD Anderson Symptom Inventory (MDASI) with the CLL module (MDASI-CLL). In the V + O arm, a relevant improvement in general health status and QOL was recorded at cycle 3, whereas improvement was delayed until cycle 8 with Chlor + O. CLL-related symptoms (measured with MDASI-CLL) were similar between the two arms, and they remained low during treatment and follow-up. The authors concluded that treatment with venetoclax-obinutuzumab was associated with an earlier improvement compared to the control arm and that no negative signals on QOL with the V + O regimen were observed [64].

### 4.3. Combination Therapy

Ibrutinib and venetoclax exert preferential anti-leukemic activity in different anatomic compartments. Ibrutinib induces a rapid shrinkage of lymphadenopathy, while venetoclax leads to a rapid clearance of peripheral blood. In an ex vivo model of CLL, Pin Lu et al. demonstrated that ibrutinib inactivated a subpopulation of CLL cells more frequently encountered in the proliferation centers of the lymph nodes, whereas venetoclax was able to induce the cell death of resting CLL cells, especially in peripheral blood [65]. Moreover, BTK inhibition was shown to enhance mitochondrial BCL2 dependence in CLL cells, favoring the killing by venetoclax [66]. Based on these biologic data, trials combining BTKi and venetoclax were designed, showing the high efficacy of this combination in all age groups [23,67,68,69].

In the GLOW trial, which included older patients (>65 years) and/or patients with comorbidities (CIRS > 6 or creatinine clearance < 70 mL/min) [23], 210 untreated CLL patients were randomized to receive 3 months of lead-in ibrutinib followed by 12 months of ibrutinib and venetoclax (I + V) or Chlor + O (6 cycles). Patients with TP53 disruptions were excluded. A similar ORR was observed (86.8% with I + V and 84.8% with Chlor + O), but CR rates were higher with I + V (38.7% vs. 11.4%). Interestingly, uMRD at the end of treatment was observed in the PB and BM in the I + V arm (54.7% and 51.9%, respectively). With a median follow-up of 27.7 months, PFS was longer with I + V than with Chlor + O (hazard ratio, 0.216; 95% confidence interval, 0.131–0.357; *p* < 0.001), with an estimated 24-month PFS rate of 84.4% with I + V vs. 44.1% with Chlor + O. The PFS advantage was evident across all the specified subgroups, including patients ≥ 65 years. With a median follow-up of 34.1 months, OS was not significantly different in the two arms.

AEs of clinical interest with I + V are summarized in Table 3. The overall incidence of grade ≥ 3 AEs was similar in the two arms (75.5% and 69.5% of patients in the I + V arm and Chlor + O arm, respectively). The administration of three cycles of ibrutinib prior to venetoclax reduced the number of patients at high risk of tumor lysis syndrome (TLS) (1.9% vs. 24.5% at baseline), and no case of TLS occurred in the I + V arm. SPM was reported in 7.5% and 9.5% of patients treated, respectively, in the I + V and Chlor + O arms. The overall number of deaths at the primary analysis was similar for the two arms; however, I + V was associated with four cardiac/sudden deaths that occurred in patients with higher CIRS and ECOG PS scores, underlining the importance of a complete cardiologic assessment before ibrutinib-based treatment [23].

## 5. Real-World Evidence

Real-world evidence (RWE) is based on real-world data (RWD) collected from a variety of sources, such as electronic health records, medical claims, databases, registries, or patient-generated data [70]. RWD can inform the population of patients not included in clinical trials, which represents >95% of patients with neoplasia in the U.S. [71].

RWE uses similar endpoints as in randomized clinical trials (RCT), with OS, time to next treatment (TTNT), and time to treatment discontinuation (TTD) representing objective measures of efficacy in observational studies [72]. That being said, it is noteworthy that RWD must be interpreted with caution due to possible selection bias, as in clinical practice, treatment selection is based on an individual patient’s characteristics at baseline [73]. 

In recent years, RWD on novel agents in CLL has highlighted significant differences between patients treated in RCT and those treated in everyday practice. Treatment discontinuation rates were higher in real-world analyses than in RTC, whereas no major differences in terms of OS were noted, including in patients treated with a reduced dose or a time-limited therapy [74,75].

Goyal et al. [76] conducted one of the larger population-based retrospective cohort studies. Among 7965 Medicare patients treated for CLL, they were able to analyze 2033 patients treated with first-line ibrutinib, with a median age of 75 years and a CCI score of 4.6. At a median follow-up of 19 months, the median OS was not reached. Ibrutinib-treated patients experienced more thrombocytopenia, bleeding, atrial fibrillation, pneumonia, and renal failure than patients treated chemo(immuno)therapy regimens for CLL. Moreover, 85.6% of ibrutinib-treated patients experienced grade ≥ 3 AEs, and overall, these data show that there is considerable susceptibility to AEs in Medicare patients with CLL in the U.S. Interestingly, 2190 patients treated with first-line ibrutinib were analyzed using a nationwide U.S. electronic health record-derived database. TTNT with ibrutinib was not significantly different in patients with a high-risk cardiovascular profile, with a TTD in all patients of 15.7 months, as compared with 11.7 and 13.7 months in patients with high AFib risk and high stroke risk, respectively [77].

Mato et al. [78] analyzed data from an electronic health record-derived database in the U.S. and reported outcomes in 1069 patients with a median age of 69 years treated in the U.S. with first-line ibrutinib and found a TTD of 38.6 months (95% CI: 33.4–42.9) and a shorter OS in patients with del(17p) than in patients without (57.7 months vs. not reached; *p* = 0.0006). 

In an unbiased nationwide survey of 747 patients with 17p/TP53 deletion and a median age of 71 years (range 32–95) treated upfront with ibrutinib, an estimated treatment persistence rate of 63.4% (95% CI 60.0–67.0%) and survival rate of 82.6% (95% CI 79.9–85.4%) were recorded at 24 months. A higher risk of treatment discontinuation was associated with age, ECOG-PS, and pre-existing heart disease. ECOG ≥ 1, age ≥ 70 years, and male sex were associated with an increased risk of death [79]. Interestingly, single- or double-hit TP53 aberrations had no impact on TTD or OS in the subgroup of 496 patients (66.4% of the total population) with similar clinicobiologic characteristics as the entire cohort. In the Italian CLL campus group report that included 100 treatment-naïve CLL patients with TP53 disruption treated with ibrutinib, the 36-month PFS and OS rates were 75% and 87%, respectively. Male gender, double-hit TP53 disruption, lack of response, and CIRS > 6 were associated with decreased OS. [80].

RWD were also reported to compare the efficacy of first-line ibrutinib and CIT. Cuneo et al. performed a matched-adjusted indirect comparison between a European cohort of unfit CLL patients treated with BR (157 patients) and a U.S. cohort of 162 patients ≥ 65 years treated with ibrutinib, excluding patients with del(17p)/TP53 aberrations [81]. Ibrutinib was associated with a significant prolongation of PFS but not of OS. In patients with advanced-stage disease, there was also a trend for OS prolongation in favor of the ibrutinib cohort. 

An indirect comparison of CIT with Chlor + O and ibrutinib was performed with the Italian CLL campus network [82]. Patients with TP53 disruptions were excluded. The cohort of patients treated with Chlor + O had a higher CIRS score, worse renal function, and a higher rate of M-IGHV. ORR were similar (87% for Chlor + O and 86% for ibrutinib), but the Chlor + O group had a higher CR and uMRD rate. The 30-month PFS rate (93% vs. 68%; *p* = 0.0061) and TTNT (97% vs. 88%, *p* = 0.0043) were significantly longer with ibrutinib. After a propensity score-matched analysis was performed to balance differences between treatment groups, the PFS and TTNT advantages in favor of ibrutinib were maintained, whereas no OS advantage was observed. In the Chlor + O group, a higher incidence of AEs than in the ibrutinib group was recorded, with 2.98 vs. 1.68 AE/month of treatment/person observed.

## 6. Cost-Effectiveness

Over the last 10 years, concerns have been raised regarding the sustainability of expenditures for new drugs in hematology, even in high-income countries. The average price of drugs appears to increase over time [83], and the usage of oral-targeted therapies in the United States was estimated to cause a 590% increase in the annual cost of therapy in CLL with respect to the CIT era [5]. Medicare spending in the U.S. from 2004 to 2020 for oral drugs in CLL increased from USD 254 million to USD 3.7 billion due to the growing number of beneficiaries and the rising costs for a 30-day supply of the first-in-class BTKi ibrutinib [84]. The rising costs of medicines increase the total out-of-pocket expense for many patients, with a possible negative impact on adherence to treatment and potentially undermining the effectiveness of therapy [85]. Interestingly, a significant proportion of U.S. patients chose the lower-cost medicine when presented with a choice between two medicines and their out-of-pocket cost [86]. Clearly, the trend of unaffordable drug prices demands action to define fair prices for new drugs to guarantee effective treatment to as many patients as possible. Initiatives for assessing the value of cancer treatment and price negotiation were recently reviewed, along with possible initiatives by oncologists and health systems to mitigate the financial burden of CLL [87,88]. In some countries, the willingness to pay (WTP) for a new drug based on its efficacy as assessed by incremental cost-effectiveness ratios (ICERs) was defined. The ICER represents the difference in cost between two different treatments divided by the difference in their effect in terms of quality-adjusted life years (QALY) gained by the new treatment compared with the standard of care. One QALY equates to one year in perfect health and represents a single number that can be compared across different types of treatments [87]. 

Independent studies that assessed ICERs and QALY in previously untreated CLL were performed for the first-in-class BTKi ibrutinib, for acalabrutinib, for V + O, and for the combination ibrutinib and venetoclax. The results of cost-effectiveness analyses conducted by health-policy institutions or by non-sponsored teams of university researchers are summarized in Table 4. These data consistently show that finite-duration treatment with V + O or with ibrutinib combined with venetoclax and continuous treatment with acalabrutinib represent cost-effective options as compared with most comparators for first-line treatment of CLL in several countries, independent of disease characteristics. Noteworthy, confidential discounts were applied in the UK, and the BTKi ibrutinib may be cost-effective only after a significant reduction in the cost of treatment in the U.S. 

A company-sponsored probabilistic analysis showed that with a willingness to pay USD 50,000/QALY gained in Canada, VEN + O has the greatest probability of being cost-effective as compared with Chlor + O, BR, ibrutinib, and acalabrutinib [89]. Likewise, acalabrutinib monotherapy showed a 59% to 73% probability of being cost-effective vs. Chlor + O at a USD 100,000-to-150,000/QALY gained in the U.S. in a company-sponsored analysis [90]. An investigator-initiated study funded by an unrestricted research grant from a company showed that with a willingness to pay EUR 23,600–35,600 per QALY, fixed-duration therapy with VEN + O was cost-effective with respect to Chlor + O, whereas the comparison of ibrutinib vs. Ven + O yielded a substantially increased incremental cost over a lifetime horizon, with an ICER of EUR 302,156/QALY [91].

Overall, these analyses are reassuring for the cost-effectiveness of target therapy according to the WTP in high-income countries and clearly support the need to take action to negotiate fair prices, especially for continuous treatment [92]. This process is especially important for CLL, whose prevalence is expected to increase due to the efficacy of new agents and the rising median age of the population in several countries [5,93,94].

## 7. Conclusions and Future Directions

The introduction of targeted agents revolutionized the approach to the treatment of CLL, regardless of risk factors or age. Indeed, BTKi and venetoclax, with or without the anti-CD20 monoclonal antibody obinutuzumab, were convincingly shown to represent relatively safe and effective agents. According to recent guidelines or expert opinion papers, they represent the preferred options for the first-line therapy of CLL [44,95,96,97]. That said, it is important to recall that a survival advantage over CIT has not been documented so far in older patients [98], due to the effectiveness of novel agents as first salvage regimens [99], and novel prognostic factors represent an area of intense investigation [100,101,102,103]. Furthermore, in the COVID-19 era, a high case-fatality rate and a poor response to vaccination were documented with fixed-duration and continuous treatment [104,105,106]. Overall, despite these improvements, the influence of racial and ethnic identity on OS in CLL is still significant, despite an encouraging possible reduction in the survival disparity between Black and White U.S. patients within the last 5 years [107].

Although a recent excellent review discussed treatment options in elderly CLL patients based on risk factors and comorbidities [108], this article provided a comprehensive description of the role of geriatric assessment along with the updated results of clinical trials and pharmacoeconomic studies. Some issues discussed in this review are relevant in everyday practice and need some perspective. 

The interpretation of the excellent results reported in studies designed for older patients should consider that inclusion criteria were heterogeneous across trials, with some investigators adopting a 65-year age cut-off and others using renal disfunction and a high cumulative illness rating scale defined for geriatric patients as inclusion criteria. In view of the widespread adoption of targeted agents, the identification of CLL-specific parameters identifying unfit patients would be highly desirable to tailor the intensity of treatment to the characteristics of each patient. It would also be very important to define parameters predicting the onset of drug-specific adverse events, i.e., cardiovascular toxicity and bleeding with BTKi, tumor lysis syndrome, infusion reactions, and neutropenia with venetoclax and obinutuzumab. 

Heterogeneous inclusion criteria and some notable exclusion criteria in clinical studies limit the transferability of efficacy data to everyday practice. Higher discontinuation rates with BTKi were reported in real-world studies [75], and the analysis of data from registries [78], or unbiased databases with 100% capture of the patient population treated with an anti-CLL drug outside of clinical trials, is of utmost importance to identify factors predicting earlier discontinuation in a real-world setting [79].

QOL is a very important issue in older patients, and, interestingly, fixed-duration treatment with V + O produced an earlier improvement compared to CIT [16]. Furthermore, relevant improvements in health-related quality of life were reported with fixed-duration treatment in older CLL patients with and without geriatric impairments [109]. BTK inhibitors may improve QOL as compared with chlorambucil and reduce the burden of AE during the first 6 months of treatment as compared with the BR regimen [54]. The results of QOL assessment are awaited for second-generation BTKi, which appears to be better tolerated than the first-in-class agent ibrutinib [56,110]. International questionnaires for assessment of health-related quality of life in CLL are available [111] and may represent an important tool for future research to guide treatment decisions in CLL, with special reference to the choice of fixed-duration or continuous treatment. 

Recent independent pharmacoeconomic analyses showed that targeted agents represented a cost-effective use of health system resources in high-income countries under confidential agreements on discounted prices [112,113,114,115,116,117,118]. Most importantly, targeted agents appeared to be dominant, i.e., more effective, and less costly than CIT in some analyses, as summarized in Table 4. Fixed duration treatment with targeted may be expected to result in significant cost reductions in a recent analysis [119].

However, it is noteworthy that the overall expenditure for new drugs in CLL is projected to increase substantially, posing the issue of sustainability. Furthermore, out-of-pocket expenses may limit accessibility to these drugs, and actions need to be undertaken by regulatory agencies to negotiate fair prices.

**Figure 1:**
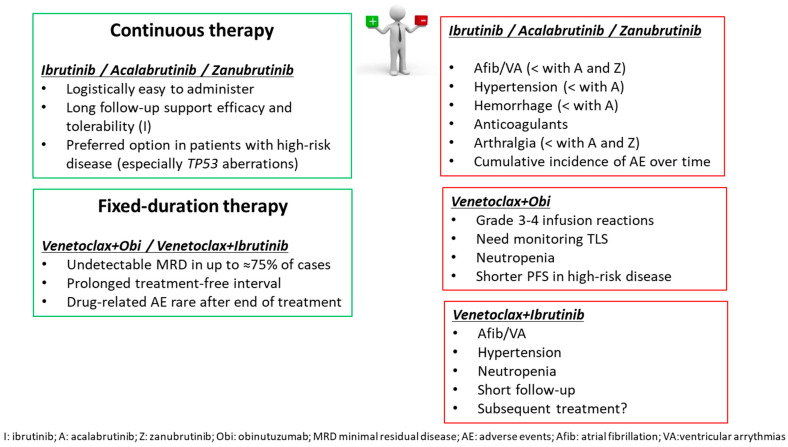
Therapy choice drivers in older patients with CLL.

Thus, today, personalized treatment of older patients with CLL is possible, and the choice of a finite-duration approach based on venetoclax-containing regimens or treatment until progression with BTKi should be discussed with every patient, taking into consideration coexisting medical conditions, logistics, and sustainability, as summarized in Figure 1.

## Figures and Tables

**Table 1:** Results of phase 3 clinical trials at the time of primary pre-planned analyses comparing the BTKi ibrutinib, acalabrutinib, and zanubrutinib, or venetoclax-containing regimens versus standard chemo(immuno)therapy in treatment-naïve older patients.

	N. of Patients	Median Age (Years)	Median Follow-Up (Months)	% TP53 Aberrations	PFS and HR (95% CI)	OS	CR/ORR (%)	Reference
BTKi vs. comparator								
IBR vs. Chlor	269	72–73	18.4	0%	18-month PFS: IBR 90%; Chlor 52%; HR 0.16 (0.09–0.28)	2-year OS: IBR 98%; Chlor 85%	4/86 2/35	[49]
IBR vs. IBR + R vs. BR	547	71	38	10%	2-year PFS: IBR 87%; IBR + R 88%; BR 74%; HR IBR 0.37 (0.25–0.56); HR IBR + R 0.40 (0.27–0.60)	2-year OS: IBR 90%; IBR + R 94%; BR 95%	7/93 12/94 26/81	[21]
IBR + O vs. Chlor + O	229	70–72	31.3	16%/20%	30-month PFS: IBR + O 77%; CHLOR + O 16%; HR 0.23 (0.15–0.37)	30-month OS: IBR + O 86%; CHLOR + O 85%	19/88 8/73	[50]
ACALA + O, ACALA, Chlor + O	535	70	28.3	9%	2-year PFS: ACALA 87%; ACALA + O 93%; CHLOR + O 47%; HR ACALA + O 0.10 (0.06–0.17) HR ACALA 0.20, (0.13–0.30)	2-year OS: ACALA 95%; ACALA + O 95%; CHOLOR + O 92%	1/85 24/94 5/79	[24]
ZANU vs. BR	479	70 years	26.2	0% *	24-month PFS: ZANU 85.5%; BR 69.5%; HR 0.42 (0.28–0.63)	24-month OS: ZANU 94.3%; BR 94.6%	7/95 15/85	[15]
Venetoclax-containing regimen vs. comparator								
V + O vs. Chlor + O	432	72–74	28.1	11.1%	24-month PFS: VO 88.2%; CHLOR + O 64.1% HR 0.35 (0.23 to 0.53)	24-month OS: VO 91.8%; CHLOR + O 93.3%	49/85 33/71	[20]
IBR + V vs. Chlor + O	211	71	27.7	4.3%	30-month PFS: IBRU + V 80.5%; CHLOR + O 35.8%; HR 0.216 (0.131 to 0.357)	NR	39/87 11/85	[23]

* a separate cohort with 111 patients and 17p deletion was reported, showing a 90% ORR with 6% CR and 24-month PFS and OS rates of 89% and 94%, respectively. HR: hazard ratio for PFS of the target agent vs. CIT; CR: complete response; ORR: overall response rate; IBR: ibrutinib; R: rituximab; CHLOR: chlorambucil; O: obinutuzumab; ACALA: acalabrutinib; ZANU: zanubrutinib; V: venetoclax; NR: not reported.

**Table 2:** Percentage of grade ≥ 3 adverse events of clinical interest at the time of primary pre-planned analyses in phase 3 clinical trials comparing standard chemo(immuno)therapy and BTKi in treatment-naïve older patients.

Trial	Median Follow-Up (Months)	AFib (*)	Hypertension	Bleeding	Infections ^	Arthralgia	Reference
Resonate-2	18.4	IBRU *6%/1.5%; CHLOR 0.7%	IBRU 4%; CHLOR 0%	IBRU 4% CHLOR 2%	8% 4%	IBRU 16% **; CHLOR 7%;	[49]
Alliance	38	IBR *17%/9%; IBR + R *14%/6% BR 3%/3%	IBR 29%; IBR + R 34%; BR 15%	IBR 2% IBR + R 4% BR 0%	20% 20% 15%	NR	[21]
iLLUMINATE	31.3	IBRU + O *12%/5%; CHLOR + O 0%	IBRU + O 4%; CHLOR + O 4%	NR	11% 5%	IBRU + O 1% CHLOR + O 0%	[50]
ACAL + O, ACAL, Chlor + O	28.3	A *4%; A + O *3%; CHLOR + O: *1%	A 2%; A + O 3%	A 2%; A + O 2%	11% 3.9% 2.4%	A 0.6%; A + O 1.1%	[24]
SEQUOIA	26.2	ZANU *3%; BR *3%	ZANU 6%; BR 5%	ZANU 3.5% BR 1.5%	3% 5%	ZANU 1%; BR 0.5%	[15]

(*) all grades/grade ≥ 3; ** all grades; ^ infections including pneumonia.

**Table 3:** Percentage of grade ≥ 3 adverse events of clinical interest with venetoclax-containing regimes at the time of primary analyses in phase 3 clinical trials in previously untreated older patients.

Trial	Median Follow-Up (Months)	Infusion Related Reactions	Tumor Lysis Syndrome	Neutropenia	Infections	AFib *	Reference
CLL14	28.1	V + O 9%; Chlor + O 10.3%	V + O 0.5%; Chlor + O 1.9%	V + O 52.8%; Chlor + O 48.1%	V + O 17.5%; Chlor + O 15.0%	NA	[20]
GLOW	27.7	NA	Ibr + V 0%; Chlor + O 5.7%	Ibr + V 34.9%; Chlor + O 49.5%	Ibr + V 12.3%; Chlor + O 8.6%	Ibr + V ^ 14%/6%; Chlor + O 1.9%/0%	[23]

(*) all grades/grade ≥ 3; IBR: ibrutinib; Chlor: chlorambucil; O: obinutuzumab; V: Venetoclax; AFib: atrial fibrillation; NA: not applicable; ^ four sudden deaths, all in patients with high comorbidities and an ECOG PS of 2.

**Table 4:** Cost-effectiveness analyses of target agents in first-line treatment of CLL.

Source/Country/ Reference	WTP/QALY	Treatment	Comparator	Target Population	ICER	Comments	Cost- Effective
NICE/U.K./114	GBP 20,000 to 30,000	V + O	Ibrutinib	17p	GBP 549,699 saved per QALY lost *	V + O results in cost saving of GBP 199,622 and QALY loss of 0.363 *	YES ^
			Chlor + O	Unsuitable for FCR/BR	NR	Dominant effect V + O vs. Chlor + O ° (more effective and less costly)	YES ^
			FR/BR	Suitable for FCR/BR	GBP 47,494 vs. FCR GBP 67,445 vs. BR per QALY gained	ICERs varied widely if the upper and lower bounds of the PFS and OS HR-CI were applied	NO
Dutch National Health Care Institute/ Holland/115	EUR 50,000	V + O	Chlor + O	Non-fit patients, uIGHV §	Incremental QALYs of 1.14 and cost saving EUR 159,276	Dominant effect (more effective and less costly); negotiation of prices recommended	YES
				Non-fit patients, mIGHV §	NR	V + O cost saving despite limited availability of data	YES
Erasmus University Rotterdam/Holland/116	EUR 20,000	V + O	Chlor + O	All patients	1.25 QALYs gained; EUR 62,316 saved	The sensitivity analyses demonstrated the robustness of these results	YES
Stanford University/ U.S.A./117	USD 150,000	Ibrutinib	Chlor + O	CLL without 17p	USD 189,000 per QALY gained	A reduction of USD 20,400 per year would be required to reach the WTP of USD 150,000	NO #
Erasmus University Rotterdam/U.K./118	GBP 20,000 to 30,000	Ibrutinib	Chlor + O	CLL	GBP 75,648 per QALY gained	An adequate discount on ibrutinib is required to make it cost-effective as per the U.K. thresholds	NO #
NICE/U.K./119		Acalabrutinib	Chlor + O	CLL unsuitable for FRC/BR, including 17p	GBP < 30,000 per QALY gained	Considering confidential discounts	YES
NICE/U.K./120	GBP 20,000 to 30,000	Ibrutinib and venetoclax	FRC/BR	CLL suitable for FRC/BR, including 17p	GBP < 30,000 per QALY gained	Considering confidential discounts	YES
			Chlor + O and V + O	Unsuitable for FRC/BR, including 17p	GBP <30,000 per QALY gained	Dominant effect vs. Chlo + O °	YES
			Acalabrutinib and ibrutinib		NR	Cost saving and a small QALY loss compared with acalabrutinib and ibrutinib	YES

WTP/QALY: Willingness to pay threshold per QALY gain; V + O: venetoclax and obinutuzumab; Chlor + O: chlorambucil and obinutuzumab; NR: not reported; uIGHV: unmutated Ig gene; mIGHV: mutated Ig gene; HR: hazard ratio; * When a drug is less effective and less costly than its comparator, the higher the ICER, the more cost-effective a treatment becomes; ° Dominant effect: more effective and less costly; ^ Provided that the companies provide the drugs according to the commercial arrangements; § CLL with 17p/TP53 mutated not included in the assessment; # unless a discount on ibrutinib is applied.

## Supplementary Materials

The following supporting information can be downloaded at: https://www.mdpi.com/article/10.3390/cancers15153859/s1, File S1: Literature Search.
