# Supplementary material for: First-Line Treatment of Older Patients with CLL: A New Approach in the Chemo-Free Era
Source: Cancers (Basel). 2023 Jul 29;15(15):3859. doi: 10.3390/cancers15153859 (PMC10417156; doi:10.3390/cancers15153859)

Literature Search

A literature search was performed to identify studies evaluating the role of targeted agents in the upfront treatment of CLL, particularly covering three areas: clinical trials, real-world data, and cost-effectiveness.

We used PubMed as the search engine, using MeSH-controlled vocabulary as follows: (("Leukemia, Lymphocytic, Chronic, B-Cell"[Mesh]) AND ("ibrutinib" [Supplementary Concept] OR "acalabrutinib" [Supplementary Concept] OR "zanubrutinib" [Supplementary Concept] OR "venetoclax" [Supplementary Concept])). Citations were restricted using the PubMed age filter (65+ years); any restriction was applied on the publication date; 458 citations were retrieved, spanning a period from Oct 2012 to March 2023. The manuscripts included in our analysis fulfilled the following inclusion criteria: i) English language; ii) full-text paper available; iiia) phase 3 clinical trial, iiib) real-world study, or iiic) cost effectiveness studies; iv) upfront therapy of CLL; and v) elderly or unfit patient population. Papers evaluating the use of CIT were excluded. The Preferred Reporting Items for Systematic Reviews and Meta-Analyses (PRISMA) guidelines (Moher D et al; Systematic reviews 4.1 (2015)) were used to report the manuscript selection process (Figure S1).

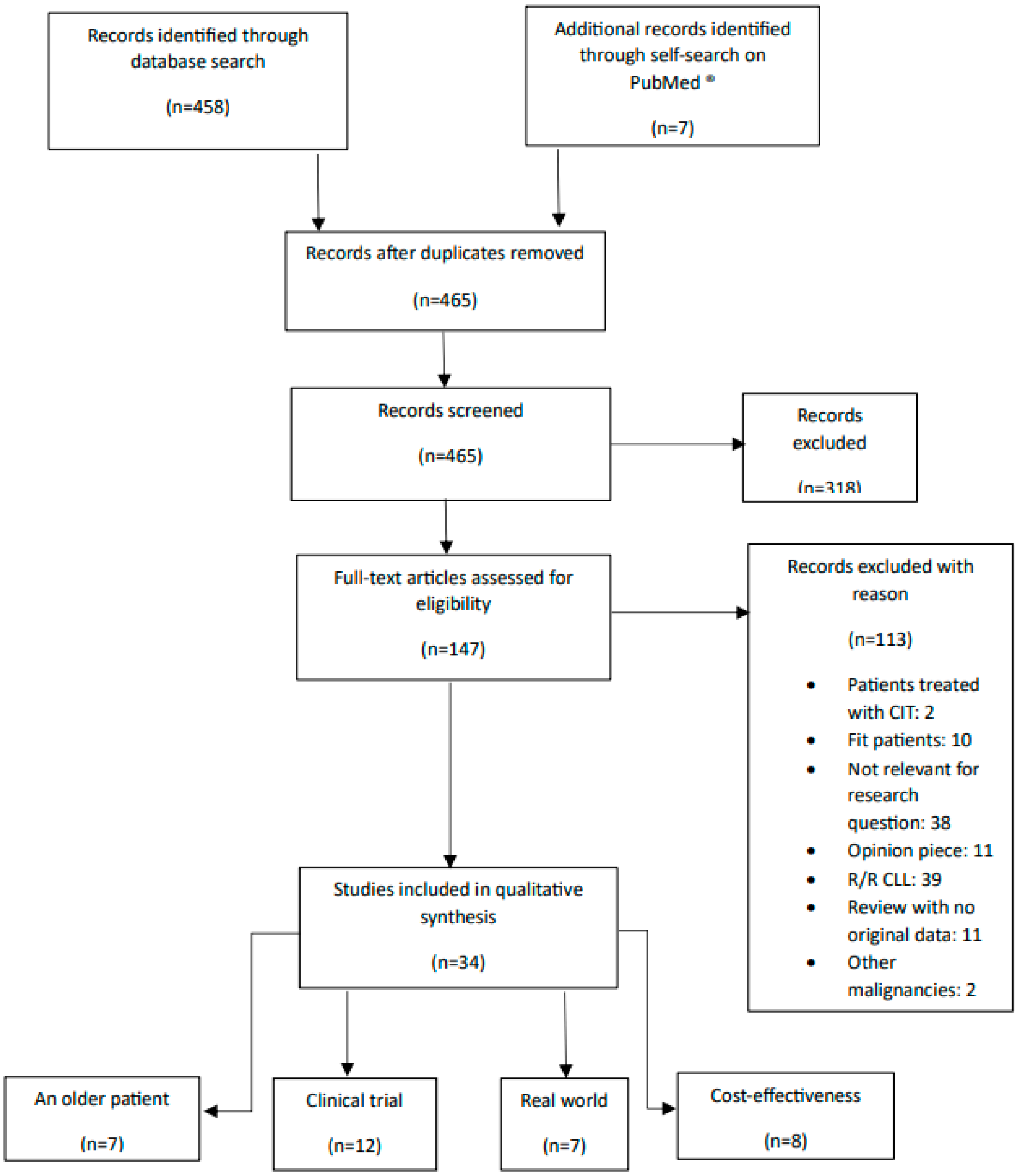

Supplement: Supplementary file 1 [file cancers-15-03859-s001.zip › cancers-2498075-supplementary.pdf]
